# The Influence of Diabetes Mellitus on Patients Undergoing Primary Total Lower Extremity Arthroplasty: A Systematic Review and Meta-Analysis

**DOI:** 10.1155/2020/6661691

**Published:** 2020-12-15

**Authors:** Wanjin Qin, Xiaoxiong Huang, Huilin Yang, Minjie Shen

**Affiliations:** Department of Orthopaedics, The First Affiliated Hospital of Soochow University, Soochow University, Suzhou 215006, China

## Abstract

**Background:**

Diabetes mellitus (DM) is a common disease that has an adverse impact on most orthopedic surgeries, and its prevalence has gradually increased in recent years. We aim to investigate the influence of DM on comorbidities and complications of patients undergoing primary total lower extremity arthroplasty.

**Methods:**

PubMed, Embase, Cochrane Library, Medline, and Web of Science were systematically searched for relevant studies published before December 2019. Demographic data, comorbidities, and postoperative complications after primary total hip arthroplasties (THA) or primary total knee arthroplasties (TKA) were assessed between DM and non-DM patients. Meta-analysis was conducted using Review Manager 5.3, and forest plots were drawn for each variable.

**Results:**

A total of 1,560,461 patients (215,916 patients with DM and 1,344,545 patients without DM) from 23 studies were included in this meta-analysis. The incidences of several preoperative comorbidities (hypertension (HTN), kidney disease, cardiac and cerebrovascular disease) were generally higher in patients with DM. Moreover, DM patients had a higher rate of postoperative complications (superficial and deep infection, deep vein thrombosis (DVT), and in-hospital mortality) compared to non-DM patients.

**Conclusions:**

DM patients were more likely to suffer from comorbidities and had a higher risk of complications in total lower extremity arthroplasty compared to non-DM patients. It is necessary to identify DM and control hyperglycemia in the perioperative period to prevent postoperative complications in patients with DM.

## 1. Introduction

Elective primary total lower extremity arthroplasty, which mainly refers to total knee and hip joint arthroplasty (TKA and THA), is a major operation frequently performed for knee and hip disease patients to reduce joint pain and increase joint mobility and function [1]. It is reported that over 500,000 patients receive lower extremity arthroplasties each year, and the demand is expected to rise to over 4 million per year before 2030 [2]. However, TKA and THA patients with certain preexisting diseases are at increased risk of postoperative complications. TKA and THA patients tend to have a higher number of comorbidities than the general population [3, 4].

DM is a chronic disease that is associated with negative outcomes after surgery. Previous studies have shown that more than 50% of DM patients have a joint disease which may require hip or knee replacement surgery. With the rising incidence of DM worldwide, the number of DM patients requiring arthroplasty is expected to increase in the future [5]. Several studies have investigated the impact of DM on the postoperative prognosis for patients undergoing TKA and THA and have indicated that DM patients have an increased rate of infection, vascular disease, and myocardial infarction compared with non-DM patients [6–8]. A meta-analysis published in 2014 reported that patients with DM had a higher risk of deep vein thrombosis, aseptic loosening, deep infection, and periprosthetic fracture after TKA [9]. Moreover, in 2013, Tsang et al. found that the incidence of infection in both nonsurgical and surgical sites was higher in DM patients compared to non-DM patients following THA [10]. Unfortunately, in recent years, no meta-analysis has been performed to investigate the effect of DM on elective primary total lower extremity arthroplasty.

The current meta-analysis was carried out to compare the comorbidities and postoperative complications between DM and non-DM patients who underwent primary TKA or THA. The intended benefits of the study are to help guide surgeons to improve treatments for patients with DM undergoing TKA and THA.

## 2. Material and Methods

### 2.1. Search Strategy

#### 2.1.1. Literature Search

According to PRISMA guidelines and statements, several literature databases, including PubMed, Embase, Cochrane Library, Medline, and Web of Science, were used to search articles involving comorbidities and postoperative complications in patients, with or without DM, undergoing elective primary TKA or THA. The search was performed for articles published before December 2019. The following Medical Subject Heading (MeSH) terms and textual words were used as the search strategy: “diabetes mellitus,” “diabetics,” “hip,” “knee,” “arthroplasty,” and “replacement.” The search terms were first based on the title and abstract, and the full text was retrieved if a decision could not be made from the summary.

#### 2.1.2. Selection Criteria

The initial selected literatures were further reviewed for inclusion according to the following uniform criteria. Two investigators assessed and selected articles independently, and all disagreements between the investigators were resolved by discussion.

Several criteria for inclusion were as follows: (1) at least two types of comorbidities or complications were reported in the study, (2) the comparison listed should contain DM patients and non-DM patients, (3) essential data could be easily extracted or calculated from the original article, (4) the language for the identified article was limited to English, and (5) full-text article is accessible.

Several criteria for exclusion were as follows: (1) the study lacked necessary variables, (2) the original data of the comparison outcomes could not be extracted, (3) animal or cell study, and (4) case reports, book chapters, review articles, summaries of experience, and cadaver studies.

#### 2.1.3. Data Extraction

Data collection was carried out independently by two authors using an identical form. Discrepancies between the two authors were resolved via discussion to reach consensus. The data extracted were as follows: year of publication, study type, demographic data, preoperative comorbidities involving hypertension (HTN), kidney, cardiac, and cerebrovascular disease, complications including DVT, infection (superficial and deep), and in-hospital mortality. T1DM, T2DM, and other secondary forms of DM (e.g., insulin-dependent DM and non-insulin-dependent DM) were not analyzed separately; all subgroups were classified as the DM group in our study.

#### 2.1.4. Quality Assessment

All selected articles were further scrutinized by two authors independently. We assessed the quality of the selected studies using the Newcastle–Ottawa Scale (NOS). A separate NOS scale was developed to evaluate the quality of the cohort and case-control studies [11]. The NOS contains eight items divided into three dimensions, including selection, comparability, and—depending on the type of study—results (cohort study) or exposure (case-control study). A total score lower than three points is considered low quality, while those achieving seven points or higher are considered high quality [12].

### 2.2. Statistical Analysis

Review Manager Version 5.3 was used to perform the meta-analysis. All comparisons were dichotomous data, and we used the odds ratio (OR) and 95% confidence intervals (CI) to conduct the statistical analysis of our variables. The sample size and number of events were extracted from the original study to calculate the OR to design the forest plot. *p* < 0.05 was set as the level of significance, and *I*^2^ was set as the index to evaluate heterogeneity. If *I*^2^ < 50%, the fixed-effected model was used because of the low heterogeneity. *I*^2^ ≥ 50% was considered a significant heterogeneity; we strived to explore possible reasons for heterogeneity, such as study design, sample size, patient selection, outcome index, and evaluation standard for each identified study. A “leave-one-out” sensitivity analysis was performed by sequentially deleting one study to determine the source of heterogeneity. After excluding each study, an analysis was performed to determine whether heterogeneity still existed; if so, the random-effect model was used [13]. When high heterogeneity was caused by a large difference in subgroups, we performed a subgroup analysis to find possible factors. Besides, we used Revman software to draw funnel plots to observe the publication bias.

## 3. Results

### 3.1. Search Results

**Figure 1:**
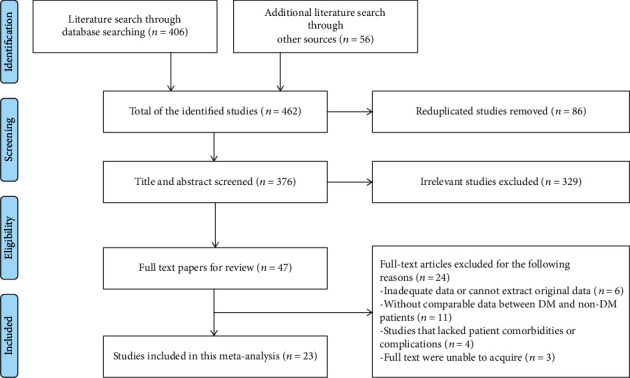
Flowchart showing the process of the literature search.

Figure 1 summarizes the details of the study identification and the process of selection. 462 articles were yielded after the initial search; then, 86 duplicates were eliminated, and then 311 of 376 records were removed based on their titles and abstracts. After downloading and identifying the full text, 42 articles without access to the inclusion criteria were excluded. Ultimately, this meta-analysis contains a total of 23 eligible studies published between 2003 and 2019 [5, 14–35].

### 3.2. Study Characteristics

In the studies identified, 18 of the 23 studies [5, 14, 15, 18, 20–29, 32–35] are cohort studies, and the remaining five [16, 17, 19, 30, 31] are case-control studies. In total, 1,560,461 cases of lower extremity total joint arthroplasty including 215,916 patients with DM and 1,344,545 patients without DM were reported. The 23 identified articles described patients undergoing primary THA or TKA. Specifically, 4 studies [16, 17, 19, 21] investigated single primary THA, 11 studies [14, 15, 18, 28–35] investigated single primary TKA, and 8 studies [5, 20, 22–27] investigated both primary TKA and THA. The detailed characteristics of each study are shown in Table 1. The NOS score of the methodological quality for each study is shown in Table 2. Among the 18 cohort studies, 13 studies [14, 15, 18, 20, 23–26, 28, 29, 32, 33, 35] had largely high quality with scores above six, 4 studies [21, 22, 27, 34] were of average quality with scores of six, and one study had a score of five points suggesting low quality. Of the five case-control studies, only one study [17] had a score of six, indicating average quality, while the other studies [16, 19, 30, 31] had scores greater than six.

### 3.3. Results of Meta-Analysis

#### 3.3.1. Comorbidities

**Figure 2:**
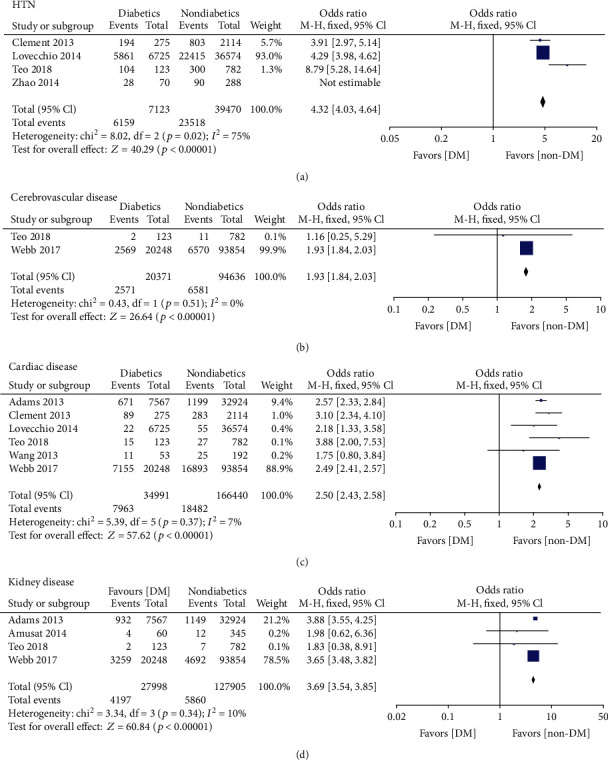
Forest plot showing the differences of comorbidities between DM patients and non-DM patients. (a) HTN. (b) Cerebrovascular disease. (c) Cardiac disease. (d) Kidney disease.

Five identified studies [18, 24, 32, 33, 35] reported the incidence of HTN before surgery in patients with or without DM. The fixed-effect model found that DM patients had significantly higher morbidity of HTN (OR = 4.26, 95% CI: 3.97, 4.57, *p* < 0.00001, *I*^2^ = 87%). However, this finding may have been influenced by high heterogeneity. After removing the study by Zhao et al. [35], heterogeneity was reduced to 75%, and sensitivity analysis and reanalysis using a random-effects model reaffirmed this significant difference (OR = 4.32, 95% CI: 4.03, 4.64, *p* < 0.00001, *I*^2^ = 75%)(Figure 2(a)). However, it should be acknowledged that heterogeneity was still present when considering this result. Two studies [32, 34] and six studies [14, 18, 24, 32–34] reported the rate of cerebrovascular disease and cardiac disease, respectively, and the fixed-effect model indicated that DM significantly increased the risk of suffering from cerebral disease (OR = 1.93, 95% CI: 1.84, 2.03, *p* < 0.00001, *I*^2^ = 0%) (Figure 2(b)) and cardiac disease (OR = 2.50, 95% CI: 2.43, 2.58, *p* < 0.00001, *I*^2^ = 7%) (Figure 2(c)). Additionally, four studies [14, 15, 32, 34] investigated the incidence of kidney disease between DM and normal patients. The fixed-effect model found a significant difference in increased incidence of kidney disease in DM patients (OR = 3.69, 95% CI: 3.54, 3.85, *p* < 0.00001, *I*^2^ = 10%) (Figure 2(d)).

#### 3.3.2. Complications

**Figure 3:**
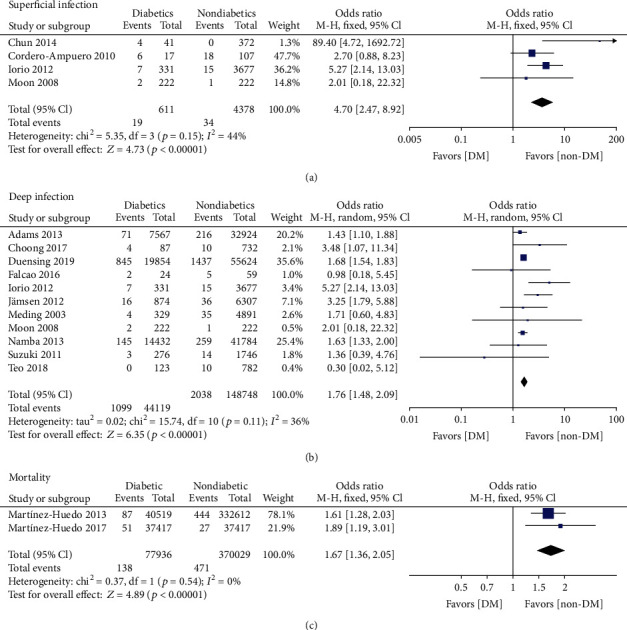
Forest plots showing the differences of infection and in-hospital mortality between DM patients and non-DM patients. (a) Superficial infection. (b) Deep infection. (c) Mortality.

**Figure 4:**
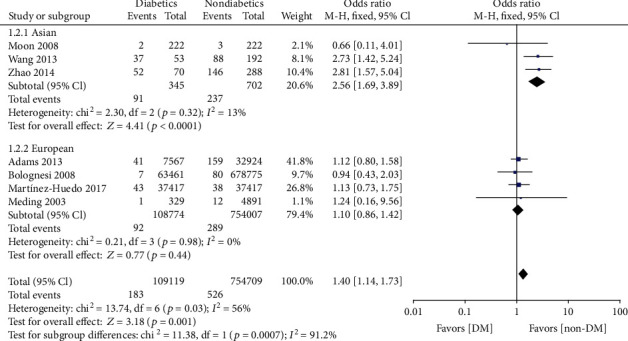
Forest plots showing the subgroup analysis of DVT.

**Figure 5:**
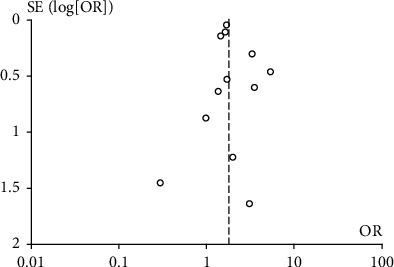
Funnel plot based on the deep infection evaluating publication bias.

Twelve articles [14, 16, 20–23, 28–32] studied the influence of DM on deep infection in patients after primary lower extremity arthroplasty. Using the fixed-effect model, we observed that patients with DM had an increased risk of deep infection (OR = 1.76, 95% CI: 1.48, 2.09, *p* < 0.00001, *I*^2^ = 36%) (Figure 3(b)). The relationship between DM and superficial infection was mentioned in five studies [17, 19, 22, 29], and the fixed-effect forest plot showed that DM was associated with a higher incidence of superficial infection (OR = 4.70, 95% CI: 2.47, 8.92, *p* < 0.00001, *I*^2^ = 44%) (Figure 3(a)). Likewise, two separated studies [26, 27] reported a significant difference in in-hospital mortality between the DM group and non-DM group (OR = 1.67, 95% CI: 1.36, 2.05, *p* < 0.00001, *I*^2^ = 0%) (Figure 3(c)). The effect of DM on DVT was reported in seven studies [5, 14, 25, 28, 29, 33, 35], and the fixed-effect forest plot showed that the risk of DVT in DM patients was 1.82 times the risk in non-DM patients (OR = 1.40, 95% CI: 1.14, 1.73, *p* = 0.001, *I*^2^ = 56%). However, because of the significant heterogeneity indicated by *I*^2^ ≥ 50%, this result should be viewed carefully. A subgroup analysis was performed based on population selection. The fixed-effect model of the Asian subgroup in three studies showed that DM significantly increased the risk of DVT without notable heterogeneity (OR = 2.56, 95% CI: 1.69, 3.89, *p* < 0.00001, *I*^2^ = 13%). The European subgroup analysis in four studies [5, 14, 25, 28, 33, 35] found no significant difference between DM patients and the non-DM patients in the European population with zero heterogeneity (OR = 1.10, 95% CI: 0.86, 1.42, *p* = 0.44, *I*^2^ = 0%) (Figure 4). A funnel plot based on the findings of deep infection was drawn to evaluate publication bias; the diagram was basically symmetric (Figure 5), indicating a low risk of publication bias in this study.

## 4. Discussion

Elective primary total knee and hip arthroplasties have achieved similarly high 10-year implant survival and overall patient satisfaction rates as the effective lower extremity surgeries. Nevertheless, there are still some patients who complain about postoperative discomfort caused by surgical sequelae, such as persistent pain, infection, DVT, and functional dysfunction [36–39]. DM, a prevalent and serious disease, has been proven to increase complication rates in patients after surgery. These poor influences partly result from higher rates of comorbidities such as hypertension, cardiac-cerebral vascular disease, and renal insufficiency in DM patients [40]. Love et al. [24] indicated that DM affected clinical prognosis after primary total low extremity arthroplasty, and that DM patients had a significantly higher rate of medical complications and revision within a month after joint replacement. As far as we know, this is the first meta-analysis investigating the adverse effect of DM on patients undergoing primary lower extremity arthroplasty.

Comorbidities are common for elderly people with DM, and 40–50% of elderly people have three or more comorbidities [41–43]. It was reported that DM is accompanied by a range of diabetes-related comorbidities, including macrovascular disease (e.g., stroke and cardiovascular disease) and microvascular disease (e.g., neuropathy and retinopathy) [44]. Another study also demonstrated that DM increased the risk of disease of the cardio-cerebral vascular and other systems, making DM the major cause of premature illness and death [45]. Our meta-analysis found several statistically significant differences in several preoperative comorbidities (HTN, kidney, cardiac and cerebrovascular disease) between DM and non-DM groups. However, since we have observed high heterogeneity in the analysis of HTN, the results require careful consideration. We performed a sensitive analysis to identify the source of high heterogeneity, and found that heterogeneity was reduced to 75% by removing the study by Zhao et al. The sample size of this study was quite small compared with the other included studies; moreover, this article was the only one showing that DM created no significant differences between the two groups. The comorbidity from DM can further develop postoperative adverse outcome after surgery in patients. Therefore, early and aggressive management of DM is required to reduce comorbidities and improve prognosis.

Deep vein thrombosis (DVT), one of the venous thromboembolism, is commonly seen after hip and knee joint replacement operation [46]. Previous studies have indicated that many DM patients undergoing total hip arthroplasty (THA) or total knee arthroplasty (TKA) have an increased risk of DVT [47–50]. A number of investigators have revealed that DM was correlated with lower levels of endogenous fibrinolysis and increased levels of procoagulant factors [51–53]. DVT has proved to originate from increased blood clotting and venous stasis as well as damage to the blood vessel wall. DM has been reported to increase endothelial damage and blood coagulability and decrease fibrinolysis [54]. In our study, subgroup analysis showed that DVT also occurs more frequently in the DM group of Asian patients, and this difference was not found in the European population. Previous studies have reported that Asian patients are more likely to suffer from DVT than European patients [55]. Perhaps, this phenomenon can be explained by the differences of clinical testing standards and the number of lower extremity arthroplasty procedures conducted. Asians have fewer lower limb surgeries than Europeans. Further, the diagnosis of DVT in Asians is based on clinical manifestations, while European countries tend to use venography, the gold standard to detect deep vein thrombosis [56]. In any case, it is necessary to perform a complete examination and control hypercoagulable states in DM patients receiving TKA or THA. Arranging relevant anticoagulation therapy and encouraging patients to exercise early can decrease the recurrence and severe complications of DVT [57].

To our knowledge, the impact of DM on mortality in patients undergoing orthopedic surgery has been controversial in recent years. Several literatures focusing on the risk of mortality after orthopedic surgery indicated that DM carried an increased risk of death, but could not reach significant difference [58, 59]. In our meta-analysis, the forest plot showed that the DM group had 138 deaths in 77,936 patients representing a rate of 0.18%, while the non-DM group had 471 death in 370,029 patients, a rate of 0.13%. The difference in mortality after lower extremity arthroplasty between the two groups was significant. However, our findings on mortality should be observed carefully since only two literatures were included. Notably, uncontrolled factors such as age, sex, comorbidities, DVT, and infection are all correlated with an increased incidence of death [5]. Also, death following total joint arthroplasty of any type is known to be an extremely uncommon event. Therefore, more research is required to investigate the relationship between DM and mortality after arthroplasty.

Surgical site infections are the most common ward infections. Deep infection around the prosthesis is one of the most serious orthopedic complications for patients and can result in many adverse effects, increased rehospitalization rates, and mortality [5, 60, 61]. Our study indicated that both superficial and deep infection appeared more frequently in DM patients, indicating that patients with DM have worse immunity. Previous research focusing on the relationship between DM and the immune system mentioned that long-term hyperglycemia had an adverse influence on the immune system due to impaired leukocyte function, which increased the risk of perioperative superficial and deep tissue infections [62]. Therefore, the rational use of antibiotics and stricter aseptic working in the perioperative period for patients with DM are essential. Additionally, an observational study by Agos et al. [63] demonstrated that controlling hyperglycemia in the perioperative period could reduce the rate of infection in patients undergoing THA and TKA. Surgeons must closely monitor glucose excursions in the perioperative period to minimize the adverse reactions caused by DM [64].

## 5. Limitations

There were some limitations to this study. Firstly, the analysis of HTN had significant heterogeneity despite the use of sensitivity analysis. Secondly, DM was commonly associated with several comorbidities, which also impact postoperative outcomes after lower extremity arthroplasty. Therefore, the potential influence of these factors may have exaggerated our results. Thirdly, the different types of DM (T1DM and T2DM) among studies and the combination of secondary forms (IDDM and NIDDM) into the DM group may have introduced some bias. Lastly, literature published in languages other than English were excluded, which may lead to inevitable publication bias. Despite these limitations, this meta-analysis was based on comparable characteristics between DM groups and non-DM groups, and the results should be verifiable.

## 6. Conclusion

DM patients were found to have more comorbidities than non-DM patients. Moreover, DM had adverse influences on patient outcomes after primary total lower extremity arthroplasty, specifically with higher risks of DVT, mortality, and superficial and deep infection. This information is useful when informing DM patients about the risk of lower extremity arthroplasty and advising patients to receive DM management during the perioperative period.

## Figures and Tables

**Table 1:** Characteristics of the included studies.

Study (year)	Country	Types	Sample size (*n*)	Male (%)	Age (y)	Surgical methods	Number of patients (DM/non-DM)	Recoded parameters
Adams et al. (2013) [14]	USA	Cohort	40 491	43% in DM and 36% in non-DM	68 in DM and 68 in non-DM	TKA	7567/32924	Cardiac disease, kidney disease, DVT, revision, deep infection
Amusat et al. (2014) [15]	Australia	Cohort	405	45% in DM and 37% in non-DM	67 in DM and 68 in non-DM	TKA	60/345	Kidney disease,
Bolognesi et al. (2008) [5]	USA	Cohort	742236	39	67	THA and TKA	63461/678775	Mortality, DVT
Choong et al. (2007) [16]	Australia	Case-control	819	46	71	THA	87/732	Deep infection
Chun et al. (2014) [17]	Korea	Case-control	413	61% in DM and 58% in non-DM	56 in DM and 51 in non-DM	THA	41/372	Superficial infection
Clement et al. (2013) [18]	France	Cohort	2389	44.4% in DM and 42.2% in non-DM	70.1 in DM and 70.4 in non-DM	TKA	275/2114	HTN, cardiac disease
Cordero-Ampuero et al. (2010) [19]	Spain	Case-control	124	NP	NP	THA	17/107	Superficial infection
Duensing et al. (2019) [20]	USA	Cohort	75478	44.2% in DM and 40.7% in non-DM	65.6 in DM and 64.7 in non-DM	TKA and THA	19854/55624	Deep infection
Falcao et al. (2016) [21]	USA	Cohort	83	25% in DM and 37.3% in non-DM	72.8 in DM and 64.3 in non-DM	THA	24/59	Deep infection
Iorio et al. (2012) [22]	USA	Cohort	4008	NR	NR	TKA and THA	331/3677	Superficial infection
Jämsen et al. (2012) [23]	Finland	Cohort	7181	36	70.6	TKA and THA	874/6307	Deep infection
Lovecchio et al. (2014) [24]	USA	Cohort	43299	42% in DM and 38.6% in non-DM	66 in DM and 68 in non-DM	TKA and THA	6725/36574	HTN, cardiac disease
Maradit Kremers et al. (2017) [25]	USA	Cohort	16085	45.8	66	TKA and THA	2911/13147	Deep infection
Martinez-Huedo et al. (2013) [27]	Spain	Cohort	373131	33.3% in DM and 33.8% in non-DM	71 in DM and 70% in non-DM	TKA and THA	40519/332612	Mortality,
Martínez-Huedo et al. (2017) [26]	Spain	Cohort	74834	37.2	71.5	TKA and THA	37417/37417	Mortality, DVT
Meding et al. (2003) [28]	USA	Cohort	3519	48% in DM and 40% in non-DM	70 in DM and 70 in non-DM	TKA	329/4891	DVT, deep infection
Moon et al. (2008) [29]	Korea	Cohort	444	10.3% in DM and 10.3% in non-DM	67.6 in DM and 67.4 in non-DM	TKA	222/222	DVT, superficial infection
Namba et al. (2013) [30]	USA	Case-control	56216	37	67.4	TKA	14432/41784	Deep infection
Suzuki et al. (2011) [31]	Japan	Case-control	2022	12.5	72	TKA	276/1746	Deep infection
Teo et al. (2018) [32]	Singapore	Cohort	905	22% in DM and 21.4% in non-DM	67.7 in DM and 65.7 in non-DM	TKA	123/782	HTN, cardiac disease, kidney disease, deep infection
Wang et al. (2013) [33]	China	Cohort	245	32.1% in DM and 28.6% in non-DM	67 in DM and 67.1 in non-DM	TKA	53/192	DVT, HTN, cardiac disease
Webb et al. (2017) [34]	USA	Cohort	114102	40.8% in DM and 36.6% in non-DM	NP	TKA	20248/93854	Cardiac disease, cerebrovascular disease, kidney disease
Zhao et al. (2014) [35]	China	Cohort	358	28.6% in DM and 34.7% in non-DM	68.09 in DM and 68.17 in non-DM	TKA	70/288	HTN, DVT

Abbreviations: DM: diabetes mellitus; HTN: hypertension; DVT: deep vein embolization; NP: not provided; TKA: total knee arthroplasties; THA: total hip arthroplasties.

**Table 2:** Quality assessment using the 9-point Newcastle-Ottawa Scale (NOS).

Study	Selection	Comparability	Outcome
Adams et al. (2013) [14]	★★★	★★	★★★
Amusat et al. (2014) [15]	★★★★	★	★★★
Bolognesi et al. (2008) [5]	★★★	★	★
Choong et al. (2007) [16]	★★★	★★	★★★
Chun et al. (2014) [17]	★★★	★	★★
Clement et al. (2013) [18]	★★★★	★	★★★
Cordero-Ampuero et al. (2010) [19]	★★★	★★	★★★
Duensing et al. (2019) [20]	★★★	★	★★★
Falcao et al. (2016) [21]	★★★	★	★★
Iorio et al. (2012) [22]	★★★★	★	★
Jämsen et al. (2012) [23]	★★★★	★	★★★
Lovecchio et al. (2014) [24]	★★★	★★	★★
Maradit Kremers et al. (2017) [25]	★★★	★★	★★★
Martinez-Huedo et al. (2013) [27]	★★★	★	★★
Martínez-Huedo et al. (2017) [26]	★★★	★★	★★★
Meding et al. (2003) [28]	★★★	★	★★★
Moon et al. (2008) [29]	★★★★	★	★★★
Namba et al. (2013) [30]	★★★★	★	★★
Suzuki et al. (2011) [31]	★★★★	★	★★
Teo et al. (2018) [32]	★★★★	★	★★
Wang et al. (2013) [33]	★★★	★	★★★
Webb et al. (2017) [34]	★★★	★	★★
Zhao et al. (2014) [35]	★★★	★★	★★★
